# Supplementary figures and images for: Impact of subjective well-being on physical frailty in middle-aged and elderly Japanese with high social isolation
Source: PLoS One. 2024 Feb 26;19(2):e0297837. doi: 10.1371/journal.pone.0297837 (PMC10896516; doi:10.1371/journal.pone.0297837)

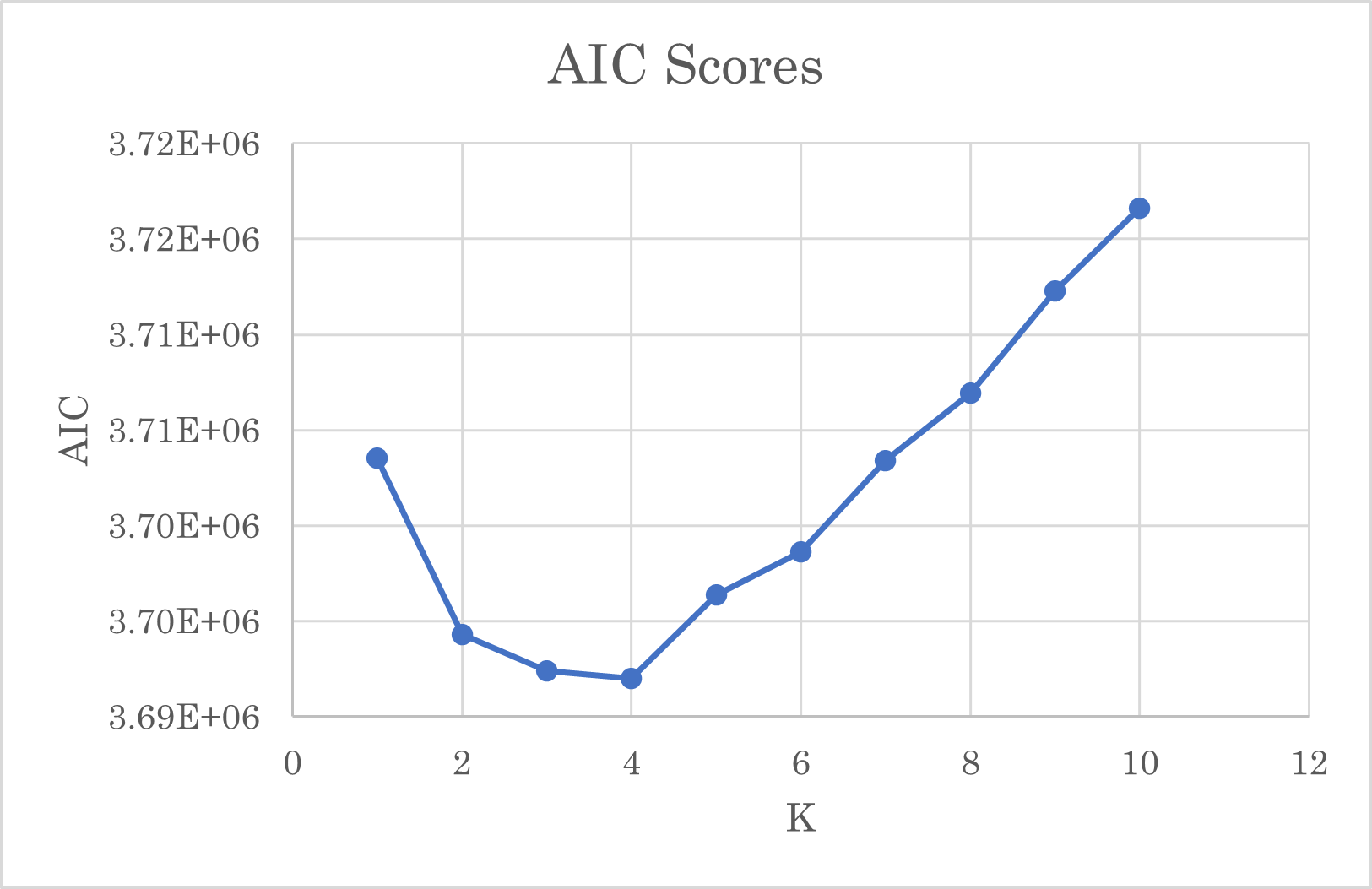

Supplement: S1 Fig — (TIF) [file pone.0297837.s001.tif]

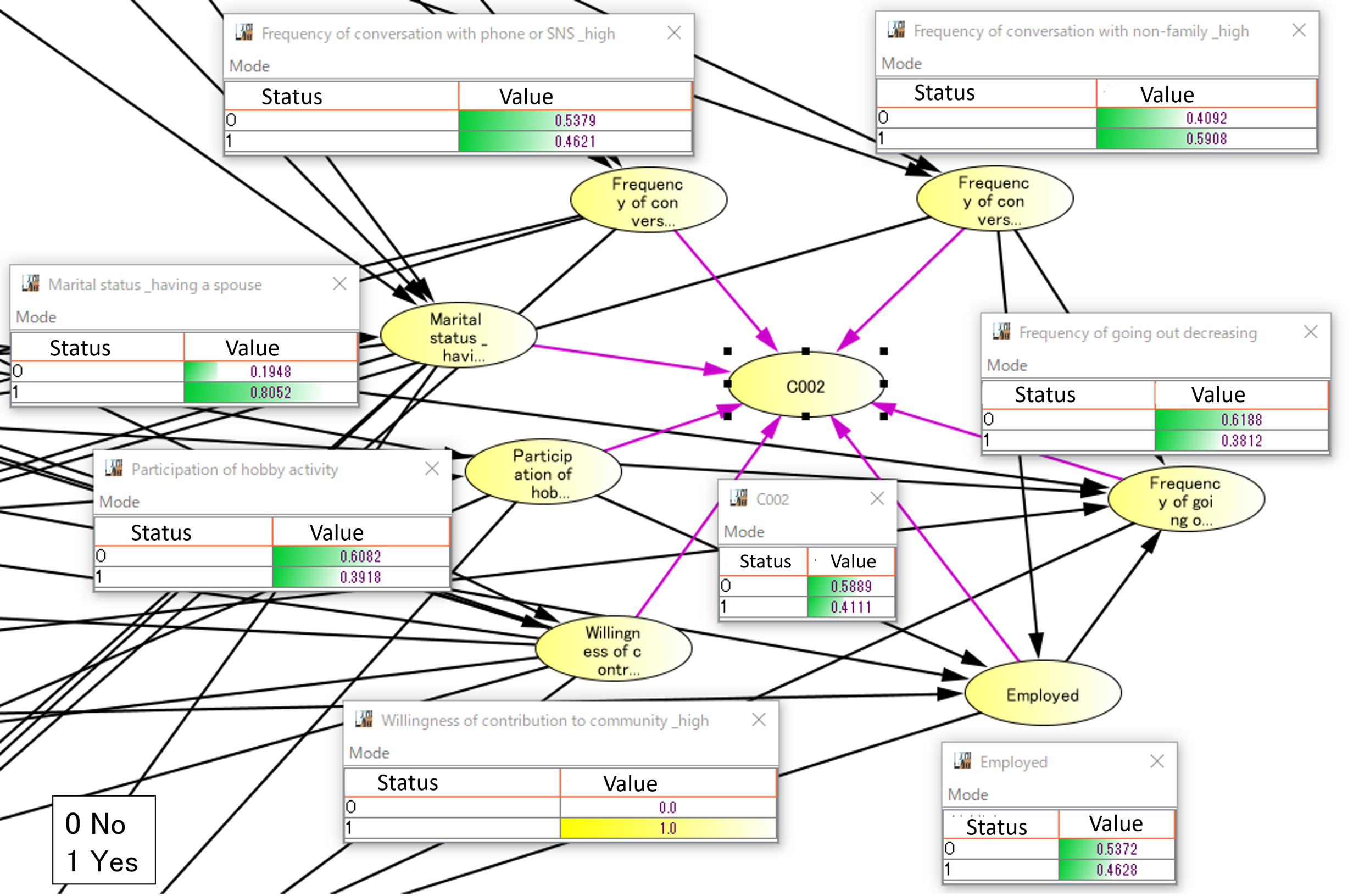

Supplement: S2 Fig — (TIF) [file pone.0297837.s002.tif]

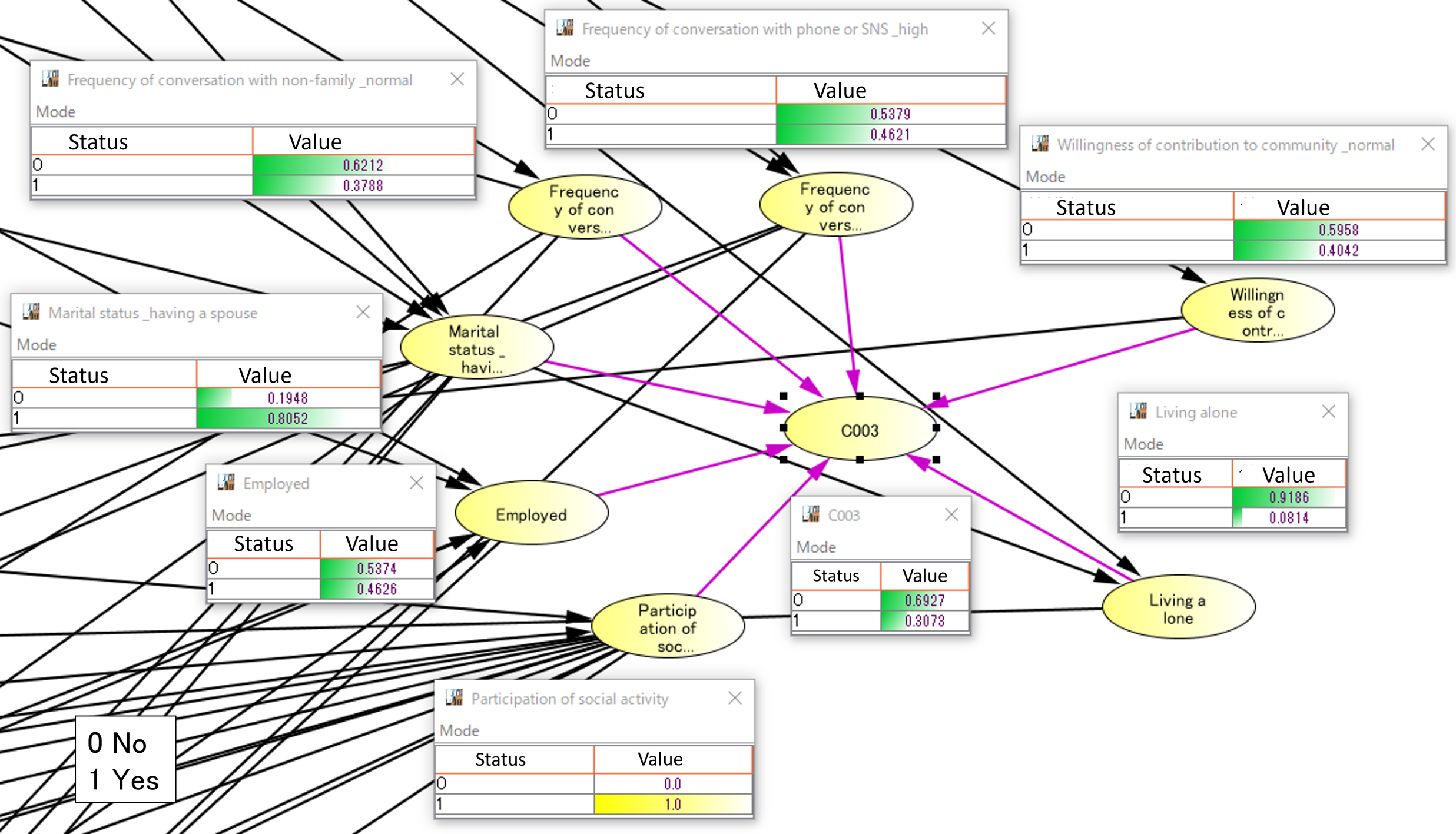

Supplement: S3 Fig — (TIF) [file pone.0297837.s003.tif]

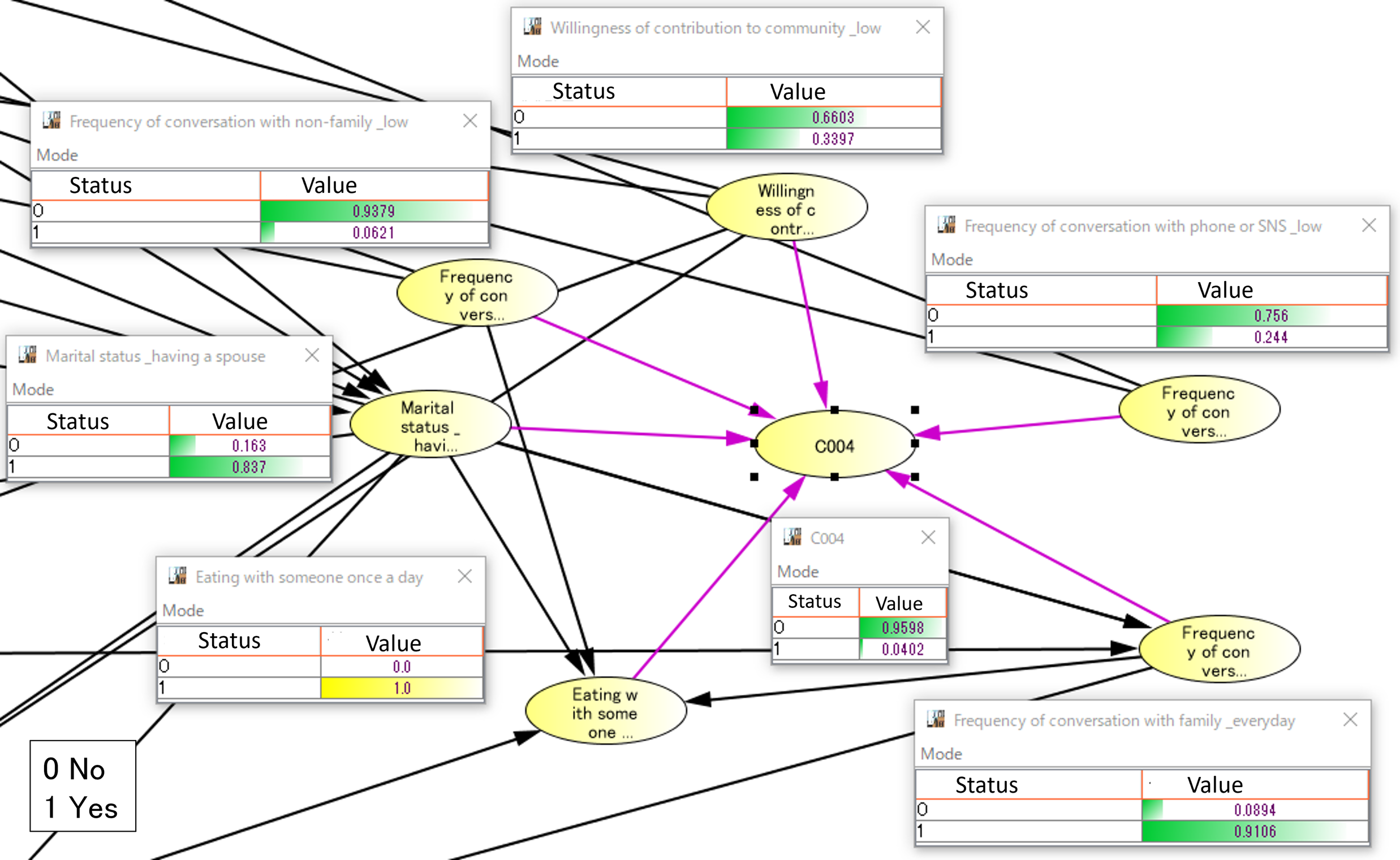

Supplement: S4 Fig — (TIF) [file pone.0297837.s004.tif]

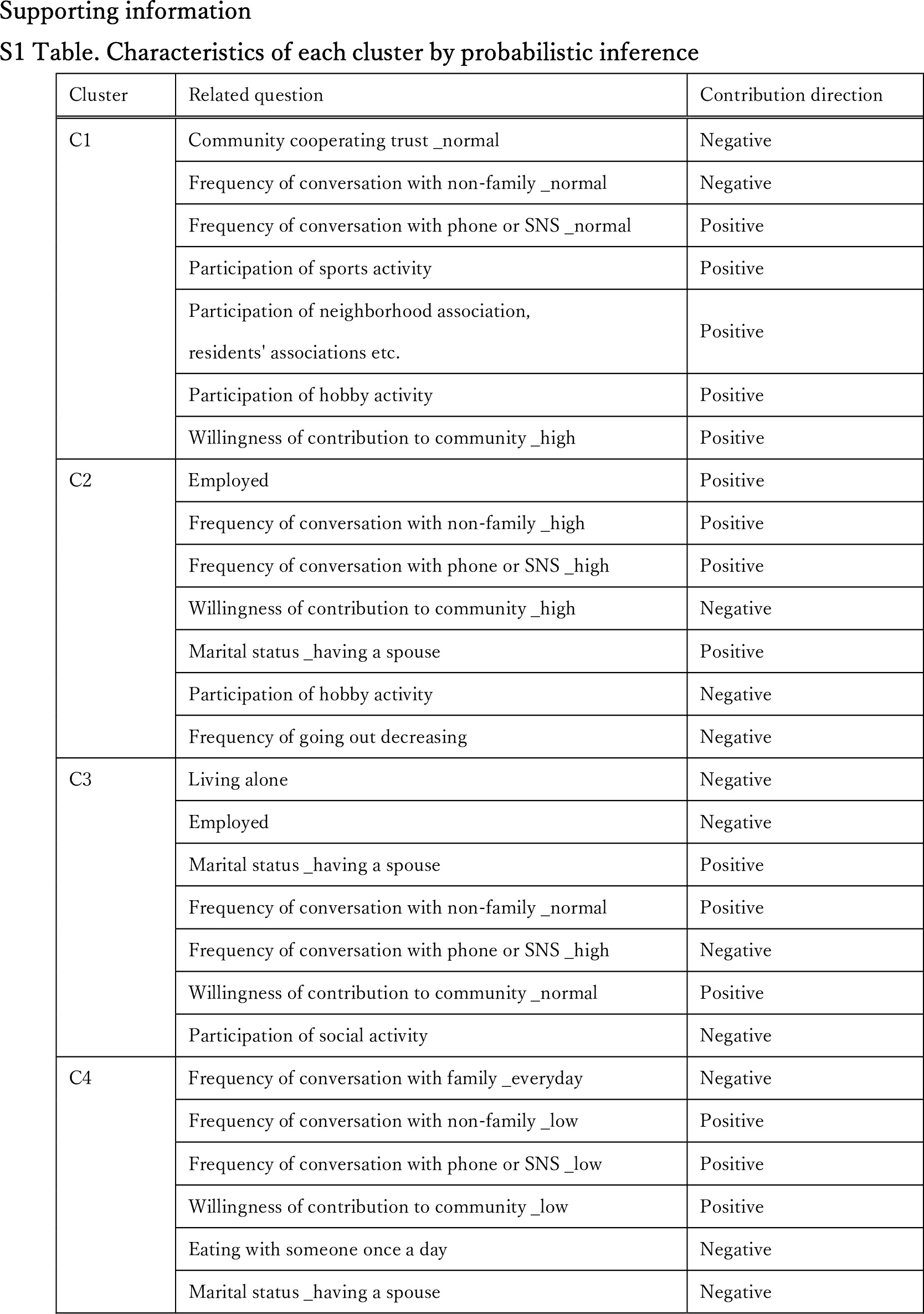

Supplement: S1 Table — (TIF) [file pone.0297837.s005.tif]
