# Supplementary material for: Impact of subjective well-being on physical frailty in middle-aged and elderly Japanese with high social isolation
Source: PLoS One. 2024 Feb 26;19(2):e0297837. doi: 10.1371/journal.pone.0297837 (PMC10896516; doi:10.1371/journal.pone.0297837)
Supplement: Attachment — Submitted filename: Response to Reviewers.docx [file pone.0297837.s006.docx]

**Responses to reviewers' comments**

**RESPONSE TO REVIEWER #1:**

Reviewer #1 comment 1:

The article identifies a relevant knowledge gap that the authors attempt to address, however, in this context, it would be important to also provide a brief description for the motivation of this study. There have been previous studies that have also associated physical frailty among older adults with a range of other health outcomes such as falls, body composition, obesity, ADL etc. Thus, a justification seems to be pertinent in this case where the authors clarify their motivation for studying social isolation with physical frailty. Adding a paragraph explicating what has been studied so far with respect to physical frailty could be beneficial focusing on the “gap” in the literature that this study aims to close.

Answer）

Thank you for your suggestion. In accordance with your comment, I have added a sentence explaining what has been studied so far regarding physical frailty.

Additions & Corrections）

Section: Introduction

> P4 L55-58

Physical frailty, the leading complaint of frailty, has been reported to increase the risk of mortality and shorten healthy life expectancy, and has been shown to be associated with other health outcomes such as gait dysfunction, falls, fractures, cognitive decline, hospitalization, nursing home placement, and quality of life [3,4].

Reviewer #1 comment 2-1:

More work needs to be done in the conclusion section. As it currently stands, the conclusion is rather limited and there is more scope to build upon the implications of the findings obtained from the study. Conclusion should be elaborated upon articulating what are keys takeaways from the article. The areas that need further strengthening are: strengths and weaknesses, implications for practice, and conclusion. The information around implication is insufficient and generates a superficial understanding.

Answer）

We agree with you. We have revised the following based on your suggestion.

Additions & Corrections)

Section: Conclusion

> P29 L412-420

In conclusion, our results suggest that subjective well-being, such as Shiawase and Ikigai, may influence physical frailty risk among middle-aged and elderly Japanese individuals with high levels of social isolation living in mountainous areas. The strength of this study is that it suggests that living with a feeling of happiness and purpose may reduce the risk of physical frailty, even among individuals with high levels of social isolation. This study provides important insights for the prevention of frailty among socially isolated individuals. On the other hand, a shortcoming of this study is that the reasons why socially isolated individuals are happy and what kind of purpose they have in life were not investigated. These aspects require further investigation.

> P30 L420-428

The study's results may serve as a reference for many other countries with aging populations. The city where participants in this study live is a typical regional city with an aging and declining population, and the average income of its citizens is about $18,000 (converted to 147.78 yen/dollar), which is in a relatively low group among all Japanese cities. In the future, many countries in the world will face aging populations. And in those countries, it is expected that the aging of the population, social isolation, and frail population will increase from rural areas compared to urban areas, resulting in widening regional disparities. The findings of this study suggest that physical frailty needs to be prevented in rural cities with aging populations, considering both the diversity of social isolation and subjective well-being in individuals.

Reviewer #1 comment 2-2:

The article should be proofread by a native English speaker. Multiple grammatical errors and awkward phrasing were found throughout the paper.

Answer）

The manuscript was edited by a native English speaker.

Reviewer #1 comment 3:

Regarding the contribution that is interesting for an international audience (or is it too focus on a specific national context)?: At present as the article stands, it would require more holistic understanding of how the notion of frailty is perceived within different country contexts, such as, lower-and-middle income countries (LMICs) as opposed to developed countries. The article should include studies from different study contexts to attract a larger number of readers. Currently, the article focuses particularly on Japan and the Japanese notion of well-being which, if constructively utilized, could be very well situated within the broader domain of aging and geriatrics to compare different studies. This would, in fact, also attract international readers and will present a higher chance of citation.

Answer）

Thank you for providing these insights. We agree with you and have incorporated the suggestion into part of the introduction and conclusion of our paper.

Additions & Corrections)

Section: Introduction

> P4 L45-49

Aging of the global population is the most important medical and sociodemographic issue that needs to be addressed not only in developed regions where the population is already aging but also in developing regions where the population is aging soon [1]. Japan has the highest percentage of population aged 65 years and older and has been called the advanced aging country; the country's actions and future are attracting global attention.

Section: Conclusion

>P30 L420-428

The results of this study can serve as a reference for other countries with an aging population. The participants in this study live in a typical regional city with an aging and declining population; the average income of its citizens is about $18,000 (converted to 147.78 yen/dollar), which is relatively low among all Japanese cities [46]. In the future, many countries will face aging populations. In these countries, it is expected that the aging population, social isolation, and frail population will increase in rural areas compared with urban areas, resulting in widening regional disparities. The findings of this study suggest that physical frailty must be prevented in rural cities with aging populations, considering both the diversity of social isolation and subjective well-being of individuals.

Reviewer #1 comment 4:

Additional comments: While the abstract is well written, however, explicit statements about the contributions of the article is largely absent. Further, the abstract does touch upon the knowledge gap but not sufficiently. The authors could try to add a few more sentences to present the gap in the literature and how the present article is particularly attempting to close that gap with the research findings.

Answer）

We wish to thank the reviewer for this comment. We have added several new sentences in the abstract section.

Additions & Corrections)

Section: Abstract

>P2 L22-23

Even those with high levels of social isolation may have different health statuses depending on the type of isolation and their subjective well-being.

>P3 L40-43

The findings reveal that even those with high level of social isolation may have a lower risk of physical frailty if their subjective well-being is high. These results will contribute to promoting the prevention of frailty in middle-aged and older adults.

**RESPONSE TO REVIEWER #2:**

Reviewer #2 comment 1:

I recommend that the author consider revising both the title and the primary aim of this study (in the introduction section) in order to better align them with the research question, conducted analyses, and the focus of the discussion section, which centers around "the relationship between subjective well-being and frailty in the high social isolation group."

Answer）

We appreciate the reviewer's comment on this point. In accordance with the reviewer's comments, the title of this article and the purpose of the introduction have been changed.

Additions & Corrections)

Section: Title

【Before】

Title: Are people with high social isolation at high risk of physical frailty? An analysis of this relationship and the impact of subjective well-being

Short title: Relationship between social isolation and physical frailty

【After】

> P1 L1-4

Title: Impact of subjective well-being on physical frailty in middle-aged and elderly Japanese with high social isolation

Short title: The relationship between physical frailty and subjective well-being in socially isolated individuals

Section: Introduction

【Before】

This study aimed to determine whether high levels of social isolation increase the risk of physical frailty among middle-aged and elderly Japanese individuals living in mountainous areas. In addition, we examined whether "Shiawase" and "Ikigai" have an effect on the frailty rate in people with high social isolation.

【After】

> P6 L91-93

This study examined the impact of subjective well-being on physical frailty in middle-aged and elderly Japanese individuals living in mountainous areas with high social isolation.

Reviewer #2 comment 2:

Within the method section, the author has indicated the existence of two data sources. To enhance clarity, please specify the variables obtained from each respective source.

Answer）

We agree that this point requires clarification, and have added the following text to the Methods.

Additions & Corrections)

Section: Methods

> P7 L118-122

The questionnaire data included demographic variables, frequency of conversations, participation in social activities, status of life activities, physical fitness, social capital in the community, and subjective well-being. The medical and long-term care insurance receipt data included the date of birth, sex, and whether the patient was certified as needing long-term care.

Reviewer #2 comment 3:

The author mentioned that “to eliminate the influence of COVID-19 as much as possible, the participants were asked to recall two time points: 2019 (before the spread of COVID-19) and 2011”. Could you kindly provide clarification on the specific questions or variables for which the participants were asked to re Additions & Corrections)

Answer）

We agree that this point requires clarification, and have added the following text to the Methods.

> P7 L115-118

The variables recalled were frequency of conversation, participation in social activities, status of life activities, physical fitness, social capital in the community, and subjective well-being (Shiawase (happiness) and Ikigai (the joy and goal of living/a life worth living)).

Reviewer #2 comment 4:

According to the data provided in the participants section, approximately 51% (2,092 out of 4,045) of the participants were excluded from the analysis. The author has acknowledged in the limitation section that this exclusion "may have biased the analysis." Could the author provide further elaboration on the implications of this bias and its potential impact on the validity and credibility of this study?

Reviewer #2 comment 5:

Furthermore, it would be better if the author could elucidate the distinctions between the excluded sample and the analytical sample, specifically concerning their sociodemographic characteristics and health status.

Answer）

We have done the additional work suggested by the reviewer and added the following text and table to the Limitation.

Additions & Corrections)

Section: Limitation

>P27 L376-383

In addition, information on individuals who had never been certified as needing long-term care between 2011 and 2019 was extracted. The sociodemographic characteristics and health statuses of the included and excluded participants are shown in Table 7. In the analyzed data (n=1,953) and the excluded data (n=1,753), the percentage of males differed by 8.3 percentage point, the average age was 4.9 years, and the rate of physical frailty was 9.8 percentage point. Among the excluded data, physical frailty was assessed in 988 samples. These differences suggest that this study analyzed a relatively healthy sample of the total data.

**Table 7. The difference of the analyzed and excluded data**

|  | n | Percentage of sex  （Male）^#^ | Percentage of age ^#^ | | | Average age (years)  （standard deviation） | Physical frailty rates ^#^ |
| --- | --- | --- | --- | --- | --- | --- | --- |
|  |  |  | under 65 | over 65,  under 75 | over 75 | all |  |
| analyzed data | 1,953 | 51.0%^a^ | 21.0%^a^ | 50.8%^a^ | 28.2%^a^ | 69.8  (8.42) | 19.6% ^a^ |
| excluded data | 1,753 | 42.7%^b^ | 9.6%^b^ | 34.7%b^b^ | 55.7%^b^ | 74.7  (8.34) | 29.4% ^b^ |

^#^ Groups with different symbols (a, b) indicate significant differences at the 5% level.

>P30 L395-401

In addition, people who have never been certified as needing long-term care from 2011 to 2019 were extracted. The sociodemographic characteristics and health status of the analyzed and excluded data are shown in Table 7. In the analyzed data (n=1,953) and the excluded data (n=1,753), the percentage of males differs by 8.3 percentage point, the average age by 4.9 years, and the rate of physical frailty by 9.8 percentage point. Of the excluded data, physical frailty was assessable in 988 samples. These differences suggest that this study is an analysis of a relatively healthy sample of the total data.

Reviewer #2 comment 6:

In my opinion, the final paragraph of the discussion section lacks coherence with the preceding paragraph. I would recommend revising it to ensure a smoother transition between the two paragraphs.

Answer）

We agree with the reviewer's comments and have added the following text before the last paragraph of the discussion section.

Additions & Corrections)

Section: Discussion

Finally, the promotion of physical frailty prevention in middle- and older-aged adults with high levels of social isolation is discussed.

Reviewer #2 comment 7:

In the introduction section, the research question was formulated as "Are all people with a high degree of social isolation at higher risk for physical frailty?" whereas, in the conclusions section, it was rephrased as "Are people with a high degree of social isolation generally at higher risk for physical frailty?" Although the two sentences share similarities, I recommend revising the one in the conclusions section to avoid any confusion.

Answer）

Thank you for your suggestion. This point has been corrected in accordance with the reviewer's comment.

Additions & Corrections)

Section: Conclusion

> P29 L409-410

The research question for this study was, ”Are all people with a high degree of social isolation at higher risk for physical frailty?.”
