# Supplementary material for: Impact of subjective well-being on physical frailty in middle-aged and elderly Japanese with high social isolation
Source: PLoS One. 2024 Feb 26;19(2):e0297837. doi: 10.1371/journal.pone.0297837 (PMC10896516; doi:10.1371/journal.pone.0297837)
Supplement: Attachment — Submitted filename: Response to Reviewers.docx [file pone.0297837.s007.docx]

**Responses to reviewers' comments**

**RESPONSE TO REVIEWER #1:**

Reviewer #1 comment 1:

1. The author(s) should also provide a descriptive statistics table with mean and standard deviation (SD) of age, *Ikigai* scores, and *Shiawase* scores, frailty, and social isolation scores (essentially the scores that have been measured for this study).

Answer）

Thank you for your suggestion. Table 3 shows that information. In addition, variable scores were appended to Table 2.

Additions & Corrections）

Section: Results

> Table 3 and P17 L236-246

The averages and standard deviations for age, *Shiawase* score, *Ikigai* score, physical frailty score, and social isolation score are shown in Table 3. Social isolation scores in this study were calculated by summing the scores of the social isolation questionnaire items (Table 2), in which 1 point was allocated for each of the following: living alone; not having a spouse; frequency of conversation with family members is not daily; not participating in any of the social activities, respectively; not eating with someone once a day; frequency of going out decreasing; not employed. In addition, the frequency of conversation with non-family and the frequency of conversation with phone or SNS, community cooperating trust, willing of contribution to community was divided into three-point scales: high, normal, and low, and assigned a score of 1, 2, and 3, respectively. In addition, the number and percentage of people in the analyzed data for employment status and education status are shown in Table 3.

Section: Methods

> Table 2

The scores of the variables were added in the table.

Reviewer #1 comment 2:

The RQ “Are all people with a high degree of social isolation at higher risk for physical frailty?” remains too broad. It still misses the ‘subjective well-being’ component which is as important part of the study. Additionally, the demographics (middle aged and elderly Japanese) could be added in the RQ as well. “All people” does not stand correct in the current phrasing and is too broad.

Answer）

We agree with you. We have revised the following based on your suggestion.

Additions & Corrections)

Section: Introduction

> P6 L90-91

Section: Conclusions

> P30 L423-424

Before revision) Are all people with a high degree of social isolation at higher risk for physical frailty?

After revision) Does subjective well-being affect physical frailty in middle-aged and elderly Japanese with high social isolation?

Reviewer #1 comment 3:

The author(s) in line 78-80 state(s) that “for physical frailty, there is a need to consider the diversity of social isolation in the prevention of physical frailty. However, no studies have yet been conducted to clarify this issue”. Considering the diversity of social participation on physical frailty, the authors could refer to the following work in the context of China:

Xie, B., & Ma, C. (2021). Effect of social participation on the development of physical frailty: Do type, frequency and diversity matter? Maturitas, 151, 48-54.

Answer）

Thank you for providing these insights. We agree with you and have incorporated the suggestion into the introduction of our paper.

Additions & Corrections)

Section: Introduction

> P5 L78-80

Before revision) Similarly for physical frailty, there is a need to consider the diversity of social isolation in the prevention of physical frailty. However, no studies have yet been conducted to clarify this issue.

After revision) Similarly for physical frailty, there is a need to consider the diversity of social isolation in the prevention of physical frailty. However, only a few studies have clarified this issue [14].

[14] Xie B, Ma C. Effect of social participation on the development of physical frailty: Do type, frequency and diversity matter? Maturitas. 2021;151: 48-54. doi: https://doi.org/10.1016/j.maturitas.2021.06.015

Reviewer #1 comment 4~6:

4. To make the study findings stronger, is it possible to supply the employment/occupation information of the participants in the descriptive statistics table? The author(s) report the study of Tanaka et al. (2020) in the context of Japan that state those who were socially isolated were highly educated, weren’t financially disadvantaged and chose to deliberately live in social isolation. However, in this study, the authors state that the participants of the city had an average income of $18000 which is not a lot, but we still do not know the employment/income status of the participants in this study. Including income and education as part of the descriptive statistics table is highly recommended. The correlation (if not causation) between income/employment and social isolation could be really interesting given the data is cross-sectional. You could maybe add some sentences of how this study findings are consistent with Tanaka et al. or not in the discussion section.

5. Although the authors do note that the purpose of social isolation has not been explored, but perhaps providing their employment/income status could be a good reference point for future studies. It could help future researchers identify whether income/education really matter when it comes to individuals isolating themselves in other country contexts.

6. On lines 341 the author(s) mention that “In our study, the number of individuals with high levels of social isolation who were intentionally socially isolated or enjoyed solitude was unclear; however, it could be inferred that there was a certain number”. Can you explicitly state the number? Or what exactly is it that you are referring to (how do you infer)? At present, it is confusing and unclear to the reader.

Answer）

We thank the reviewer for these comments. Comments 4 through 6 were similar and were therefore answered together in this section.

In our study, 7.3% of participants with high levels of social isolation were highly educated (college graduates or higher). However, we avoided discussing this study because we lacked data (income and economic status) to examine its consistency with the study by Tanaka et al. (2020).

Additions & Corrections)

Section: Results

> Table 3 and P17 L236-246

The number and percentage of people in the analyzed data for employment status and education status are shown in Table 3.

(Income data was not obtained and could not be displayed.)

Section: Discussion

> P26 L350-355

Before revision) In our study, the number of individuals with high levels of social isolation who were intentionally socially isolated or enjoyed solitude was unclear; however, it could be inferred that there was a certain number.

After revision) In our study, the number of individuals with high levels of social isolation who were intentionally socially isolated or enjoyed solitude was unclear; however, it could be inferred that there was a certain number of them. Because among the participants with high social isolation, the percentages of those with high *Shiawase* and *Ikigai* scores were 42.4% (n=103) and 48.4% (n=119), respectively. These people may be intentionally socially isolated or may enjoy solitude.
